# Sensitivity Analysis on Odds Ratios

**DOI:** 10.1093/aje/kwad137

**Published:** 2023-06-13

**Authors:** Kalle Leppälä

**Keywords:** Cornfield conditions, Cornfield inequalities, mediant inequality, odds ratio, sensitivity analysis

## Abstract

The classical Cornfield inequalities state that if a third confounding variable is fully responsible for an observed association between the exposure and the outcome variables, then the association between both the exposure and the confounder, and the confounder and the outcome, must be at least as strong as the association between the exposure and the outcome, as measured by the risk ratio. The work of Ding and VanderWeele on assumption-free sensitivity analysis sharpens this bound to a bivariate function of the 2 risk ratios involving the confounder. Analogous results are nonexistent for the odds ratio, even though the conversion from odds ratios to risk ratios can sometimes be problematic. We present a version of the classical Cornfield inequalities for the odds ratio. The proof is based on the mediant inequality, dating back to ancient Alexandria. We also develop several sharp bivariate bounds of the observed association, where the 2 variables are either risk ratios or odds ratios involving the confounder.

In the classic 1959 paper (1), Cornfield et al. dealt a deathblow to Fisher’s constitutional hypothesis that the observed association between cigarette smoking and lung cancer is noncausal, and gave birth to the field of sensitivity analysis (2). The key idea derived from Cornfield’s work is that if a strong observed association was merely due to confounding, the confounding variable needs to be strongly associated with both the exposure and the outcome.

Precisely, let the risk ratio (RR) between binary random variables *X* and *Y* be defined as:

$$ \mathrm{R}{\mathrm{R}}_{XY}=\frac{\mathbb{P}\left(Y|X\right)}{\mathbb{P}\left(Y|\overline{X}\right)}. $$

Make the assumption that *X* and *Y* are independent conditional on a third binary random variable *Z*, which is not negatively associated with either *X* or *Y*:

(1) \begin{equation*} \left(X\perp\!\!\! \perp Y\right)\mid Z,\quad\left\{\begin{array}{ll}\mathrm{R}{\mathrm{R}}_{XZ}\ge 1,& \\ {}\mathrm{R}{\mathrm{R}}_{ZY}\ge 1.& \end{array}\right. \end{equation*}

Note that the second assumption can always be satisfied by recoding the variables *X* or *Y*, and that as a consequence $\mathrm{R}{\mathrm{R}}_{XY}\ge 1$ as well. The following result is often referred to as the classical Cornfield inequalities (3), despite only the first half being proven by Cornfield (1, 4).

Theorem 1. Under Assumption 1*,*

$$\mathrm{R}{\mathrm{R}}_{XY}\le \mathrm{R}{\mathrm{R}}_{XZ},\qquad\mathrm{R}{\mathrm{R}}_{XY}\le \mathrm{R}{\mathrm{R}}_{ZY}.$$

Even though Theorem 1 is stated in terms of $\mathrm{R}{\mathrm{R}}_{XZ}$ and $\mathrm{R}{\mathrm{R}}_{ZY}$, *X* does not need to be a cause of *Z* or *Z* of *Y*, because the risk ratio is a measure of association, not causation. Assumption 1 is valid in many causal graphs; for example, in a fork where *Z* is a common cause of *X* and *Y*, or in a chain where *Z* is a mediator between *X* and *Y* (or between *Y* and *X*). It might feel more “natural” to use $\mathrm{R}{\mathrm{R}}_{ZX}$ instead of $\mathrm{R}{\mathrm{R}}_{XZ}$ in a fork, for example, but a result like Theorem 1 where $\mathrm{R}{\mathrm{R}}_{XY}$ is bounded by a function of $\mathrm{R}{\mathrm{R}}_{ZX}$ is not possible. The reason is that no matter how large $\mathrm{R}{\mathrm{R}}_{XY}$ is, $\mathrm{R}{\mathrm{R}}_{ZX}$ can be arbitrarily close to 1.

Another way to formulate Theorem 1 is $\mathrm{R}{\mathrm{R}}_{XY}\le \min \left(\mathrm{R}{\mathrm{R}}_{XZ},\mathrm{R}{\mathrm{R}}_{ZY}\right)$. A natural question is to ask how the maximum function behaves, and the answer is that it is the upper bound of the so-called E-value $\mathrm{R}{\mathrm{R}}_{XY}+\sqrt{\mathrm{R}{\mathrm{R}}_{XY}\left(\mathrm{R}{\mathrm{R}}_{XY}-1\right)}\le \max \left(\mathrm{R}{\mathrm{R}}_{XZ},\mathrm{R}{\mathrm{R}}_{ZY}\right)$ (5). That is, under Assumption 1 not only do both $\mathrm{R}{\mathrm{R}}_{XZ}$ and $\mathrm{R}{\mathrm{R}}_{ZY}$ have to be at least $\mathrm{R}{\mathrm{R}}_{XY}$; in addition, at least one of them has to be even bigger. A second natural question is to ask whether the minimum function is optimal, and the answer is negative. Ding and VanderWeele (6) have shown that the sharp bound of $\mathrm{R}{\mathrm{R}}_{XY}$ using both variables $\mathrm{R}{\mathrm{R}}_{XZ}$ and $\mathrm{R}{\mathrm{R}}_{ZY}$ is

(2) \begin{equation*} \mathrm{R}{\mathrm{R}}_{XY}\le \frac{\mathrm{R}{\mathrm{R}}_{XZ}\mathrm{R}{\mathrm{R}}_{ZY}}{\mathrm{R}{\mathrm{R}}_{XZ}+\mathrm{R}{\mathrm{R}}_{ZY}-1}. \end{equation*}

Another popular measure of the strength of association between binary variables is the odds ratio (OR):

$$ \mathrm{O}{\mathrm{R}}_{XY}=\frac{\mathbb{P}\left(Y|X\right)\mathbb{P}\left(\overline{Y}|\overline{X}\right)}{\mathbb{P}\left(Y|\overline{X}\right)\mathbb{P}\left(\overline{Y}|X\right)}. $$

The odds ratio arises naturally in the context of logistic regression or case-control design. In some situations where the outcome is rare, the risk ratio and the odds ratio are very close, and a square root conversion has been suggested for other situations (7). To do a precise conversion, however, one needs some additional parameter, such as the prevalence among the nonexposed. The additional parameter can introduce additional uncertainty, in underdiagnosed disease in particular. It would therefore be preferable to use results explicitly designed for the odds ratio rather than converting to risk ratios and using Cornfield’s inequalities or inequality (2).

In this work, we derive an odds ratio analogue of the classical Cornfield inequalities using the mediant inequality dating back to ancient Alexandria. We also compile a complete collection of 2-variable bounds analogous to inequality (2), where some of the 3 risk ratios are odds ratios instead. One such bound by Lee (8) already exists in the literature. Many combinations of risk and odds ratios do not admit a bound, and there is also symmetry in play, so we present 4 original results (2 of which are almost trivial corollaries).

For bounds involving risk differences, see Ding and VanderWeele (3).

## RESULTS

### Bounding with a single ratio

The proof of Theorem 1 will serve as a template for original results.

By the law of total probability and Assumption 1,

(3) \begin{equation*} \mathrm{R}{\mathrm{R}}_{XY}=\frac{\mathbb{P}\left(Y|X\right)}{\mathbb{P}\left(Y|\overline{X}\right)}=\frac{\mathbb{P}\left(Y|Z\right)\mathbb{P}\left(Z|X\right)+\mathbb{P}\left(Y|\overline{Z}\right)\mathbb{P}\left(\overline{Z}|X\right)}{\mathbb{P}\left(Y|Z\right)\mathbb{P}\left(Z|\overline{X}\right)+\mathbb{P}\left(Y|\overline{Z}\right)\mathbb{P}\left(\overline{Z}|\overline{X}\right)}. \end{equation*}

Cornfield calls the first inequality of Theorem 1 “rather obvious” and derives it algebraically (1, Appendix A) from Ding and VanderWeele (3). We wish to give this classical inequality a classical proof.

Lemma 2. Inequality. If $bd>0$*,* then

$$ \min \kern0em \left(\frac{a}{b},\frac{c}{d}\right)\le \frac{a+c}{b+d}\le \max \kern0em \left(\frac{a}{b},\frac{c}{d}\right). $$

Special cases of the mediant inequality were already known by Plato and Euclid, but the first known formal proof is given by Pappus of Alexandria (9, 10). The validity of the mediant inequality is easy to see geometrically: The slope of the sum vector is between the slopes of the summands. Applying the mediant inequality to equation 3 immediately gives

$$ \mathrm{R}{\mathrm{R}}_{XY}\le \max \left(\mathrm{R}{\mathrm{R}}_{XZ},\mathrm{R}{\mathrm{R}}_{X\overline{Z}}\right)=\mathrm{R}{\mathrm{R}}_{XZ}. $$

Schlesselman (4) proves the second classical Cornfield inequality by writing equation 3 as

$$ \mathrm{R}{\mathrm{R}}_{XY}=\frac{\mathrm{R}{\mathrm{R}}_{ZY}\mathbb{P}\left(Z|X\right)+1-\mathbb{P}\left(Z|X\right)}{\mathrm{R}{\mathrm{R}}_{ZY}\mathbb{P}\left(Z|\overline{X}\right)+1-\mathbb{P}\left(Z|\overline{X}\right)}, $$

and maximizing the numerator and minimizing the denominator at the point $\mathbb{P}\left(Z|X\right)=1$, $\mathbb{P}\left(Z|\overline{X}\right)=0$.

For the odds ratio, the law of total probability and Assumption 1 give

$$ \mathrm{O}{\mathrm{R}}_{XY}=\frac{\mathbb{P}\left(Y|X\right)\mathbb{P}\left(\overline{Y}|\overline{X}\right)}{\mathbb{P}\left(Y|\overline{X}\right)\mathbb{P}\left(\overline{Y}|X\right)} $$

(4) \begin{align*} \notag &=\frac{\mathbb{P}\left(Y|Z\right)\mathbb{P}\left(Z|X\right)+\mathbb{P}\left(Y|\overline{Z}\right)\mathbb{P}\left(\overline{Z}|X\right)}{\mathbb{P}\left(Y|Z\right)\mathbb{P}\left(Z|\overline{X}\right)+\mathbb{P}\left(Y|\overline{Z}\right)\mathbb{P}\left(\overline{Z}|\overline{X}\right)}\\&\quad\times \frac{\mathbb{P}\left(\overline{Y}|Z\right)\mathbb{P}\left(Z|\overline{X}\right)+\mathbb{P}\left(\overline{Y}|\overline{Z}\right)\mathbb{P}\left(\overline{Z}|\overline{X}\right)}{\mathbb{P}\left(\overline{Y}|Z\right)\mathbb{P}\left(Z|X\right)+\mathbb{P}\left(\overline{Y}|\overline{Z}\right)\mathbb{P}\left(\overline{Z}|X\right)}. \end{align*}

Using the mediant inequality (Lemma 2) to both fractions yields

$$\mathrm{O}{\mathrm{R}}_{XY}\le \max \left(\mathrm{R}{\mathrm{R}}_{XZ},\mathrm{R}{\mathrm{R}}_{X\overline{Z}}\right)\times \max \left(\mathrm{R}{\mathrm{R}}_{\overline{X}Z},\mathrm{R}{\mathrm{R}}_{\overline{X}\overline{Z}}\right)=\mathrm{O}{\mathrm{R}}_{XZ}.$$

On the other hand, we can rearrange equation 4 into

\begin{align*} \notag&\frac{\mathbb{P}\left(Y|Z\right)\mathbb{P}\left(Z|X\right)+\mathbb{P}\left(Y|\overline{Z}\right)\mathbb{P}\left(\overline{Z}|X\right)}{\mathbb{P}\left(\overline{Y}|Z\right)\mathbb{P}\left(Z|X\right)+\mathbb{P}\left(\overline{Y}|\overline{Z}\right)\mathbb{P}\left(\overline{Z}|X\right)}\\ &\quad \times \frac{\mathbb{P}\left(\overline{Y}|Z\right)\mathbb{P}\left(Z|\overline{X}\right)+\mathbb{P}\left(\overline{Y}|\overline{Z}\right)\mathbb{P}\left(\overline{Z}|\overline{X}\right)}{\mathbb{P}\left(Y|Z\right)\mathbb{P}\left(Z|\overline{X}\right)+\mathbb{P}\left(Y|\overline{Z}\right)\mathbb{P}\left(\overline{Z}|\overline{X}\right)}. \end{align*}

This time the 2 applications of the mediant inequality (Lemma 2) give

\begin{align*} \mathrm{O}{\mathrm{R}}_{XY}\!&\le\! \max \left(\!\frac{\mathbb{P}\left(Y|Z\right)}{\mathbb{P}\left(\overline{Y}|Z\right)},\frac{\mathbb{P}\left(Y|\overline{Z}\right)}{\mathbb{P}\left(\overline{Y}|\overline{Z}\right)}\!\right)\times \max \left(\!\frac{\mathbb{P}\left(\overline{Y}|Z\right)}{\mathbb{P}\left(Y|Z\right)},\frac{\mathbb{P}\left(\overline{Y}|\overline{Z}\right)}{\mathbb{P}\left(Y|\overline{Z}\right)}\!\right)\\&=\mathrm{O}{\mathrm{R}}_{ZY}. \end{align*}

We have shown analogues of the classical Cornfield inequalities for the odds ratio.

Theorem 3. Under Assumption 1*,*

$$\mathrm{O}{\mathrm{R}}_{XY}\le \mathrm{O}{\mathrm{R}}_{XZ},\qquad\mathrm{O}{\mathrm{R}}_{XY}\le \mathrm{O}{\mathrm{R}}_{ZY}.$$

### Bounding with 2 ratios

We set out to complete the works of Ding and VanderWeele (6) and Lee (8) with an exhaustive collection of formulae bounding a risk or odds ratio between *X* and *Y* with any pair of riskco or odds ratios between *X* and *Z* and between *Z* and *Y*. Not all combinations admit a bound, however.

### Bounding a risk ratio

It is not possible to bound $\mathrm{R}{\mathrm{R}}_{XY}$ with $\mathrm{R}{\mathrm{R}}_{ZX}$ or $\mathrm{R}{\mathrm{R}}_{YZ}$, so we only have 4 combinations, 2 of which are existing results (Theorems 4 and6) and 2 their immediate corollaries (Theorems 5 and7). The bounds are sharp.

Theorem 4. By Ding and VanderWeele (6). Under Assumption 1,

$$ \mathrm{R}{\mathrm{R}}_{XY}\le \frac{\mathrm{R}{\mathrm{R}}_{XZ}\mathrm{R}{\mathrm{R}}_{ZY}}{\mathrm{R}{\mathrm{R}}_{XZ}+\mathrm{R}{\mathrm{R}}_{ZY}-1}. $$

Theorem 5. Under Assumption 1*,*

$$ \mathrm{R}{\mathrm{R}}_{XY}\le \frac{\mathrm{R}{\mathrm{R}}_{XZ}\mathrm{O}{\mathrm{R}}_{ZY}}{\mathrm{R}{\mathrm{R}}_{XZ}+\mathrm{O}{\mathrm{R}}_{ZY}-1}. $$

Proof. Define and differentiate

$$f(r)=\frac{\mathrm{R}{\mathrm{R}}_{XZ}r}{\mathrm{R}{\mathrm{R}}_{XZ}+r-1},\qquad{f}^{\prime }(r)=\frac{\mathrm{R}{\mathrm{R}}_{XZ}^2-1}{{\left(\mathrm{R}{\mathrm{R}}_{XZ}+r-1\right)}^2}.$$

The claim follows from $\mathrm{R}{\mathrm{R}}_{XZ}>1$, Theorem 4, and $\mathrm{R}{\mathrm{R}}_{ZY}<\mathrm{O}{\mathrm{R}}_{ZY}$.

Theorem 6. By Lee (8). Under Assumption 1,

$$ \mathrm{R}{\mathrm{R}}_{XY}\le {\left(\frac{\sqrt{\mathrm{O}{\mathrm{R}}_{XZ}\mathrm{R}{\mathrm{R}}_{ZY}}+1}{\sqrt{\mathrm{O}{\mathrm{R}}_{XZ}}+\sqrt{\mathrm{R}{\mathrm{R}}_{ZY}}}\right)}^2. $$

Theorem 7. Under Assumption 1*,*

$$ \mathrm{R}{\mathrm{R}}_{XY}\le {\left(\frac{\sqrt{\mathrm{O}{\mathrm{R}}_{XZ}\mathrm{O}{\mathrm{R}}_{ZY}}+1}{\sqrt{\mathrm{O}{\mathrm{R}}_{XZ}}+\sqrt{\mathrm{O}{\mathrm{R}}_{ZY}}}\right)}^2. $$

Proof. Define and differentiate

\begin{align*} f(r)&={\left(\frac{\sqrt{\mathrm{O}{\mathrm{R}}_{XZ}}r+1}{\sqrt{\mathrm{O}{\mathrm{R}}_{XZ}}+r}\right)}^2,\\{f}^{\prime }(r)&=\frac{2\left(\sqrt{\mathrm{O}{\mathrm{R}}_{XZ}}r+1\right)\left(\mathrm{O}{\mathrm{R}}_{XZ}-1\right)}{{\left(\sqrt{\mathrm{O}{\mathrm{R}}_{XZ}}+r\right)}^3}. \end{align*}

The claim follows from $\sqrt{\mathrm{O}{\mathrm{R}}_{XZ}}>1$, Theorem 6, and $\sqrt{\mathrm{R}{\mathrm{R}}_{ZY}}<\sqrt{\mathrm{O}{\mathrm{R}}_{ZY}}$.

### Bounding an odds ratio

It is not possible to bound $\mathrm{O}{\mathrm{R}}_{XY}$ with risk ratios between *X* and *Z* and between *Z* and *Y*. Theorem 8 uses a familiar function of $\mathrm{O}{\mathrm{R}}_{XZ}$ and $\mathrm{O}{\mathrm{R}}_{ZY}$. Theorem 9 provides a bound as a function of $\mathrm{R}{\mathrm{R}}_{XZ}$ and $\mathrm{O}{\mathrm{R}}_{ZY}$, and by the symmetry of the odds ratio can also be used for $\mathrm{O}{\mathrm{R}}_{XZ}$ and $\mathrm{R}{\mathrm{R}}_{YZ}$. For the combination of $\mathrm{O}{\mathrm{R}}_{XZ}$ and $\mathrm{R}{\mathrm{R}}_{ZY}$, and by symmetry $\mathrm{R}{\mathrm{R}}_{ZX}$ and $\mathrm{O}{\mathrm{R}}_{ZY}$, the bound of Theorem 3 is already sharp.

Theorem 8. Under Assumption 1*,*

$$ \mathrm{O}{\mathrm{R}}_{XY}\le {\left(\frac{\sqrt{\mathrm{O}{\mathrm{R}}_{XZ}\mathrm{O}{\mathrm{R}}_{ZY}}+1}{\sqrt{\mathrm{O}{\mathrm{R}}_{XZ}}+\sqrt{\mathrm{O}{\mathrm{R}}_{ZY}}}\right)}^2. $$

Proof. Denote $\mathbb{P}\left(Z|\overline{X}\right)=r$ and $\mathbb{P}\left(Y|\overline{Z}\right)=s$, which implies

$$\left\{\begin{array}{ll}\mathbb{P}\left(Z|X\right)& =\frac{r\mathrm{O}{\mathrm{R}}_{XZ}}{r\left(\mathrm{O}{\mathrm{R}}_{XZ}-1\right)+1},\quad0<r<1,\\[5pt] {}\mathbb{P}\left(Y|Z\right)& =\frac{s\mathrm{O}{\mathrm{R}}_{ZY}}{s\left(\mathrm{O}{\mathrm{R}}_{ZY}-1\right)+1}, \quad 0<s<1.\end{array}\right.$$

Plugging this into equation 4 gives

$$ \mathrm{O}{\mathrm{R}}_{XY}=f\left(r,s\right)=\frac{\left(\mathrm{O}{\mathrm{R}}_{XZ}\mathrm{O}{\mathrm{R}}_{ZY}r+g\left(r,s\right)\right)\left(r+g\left(r,s\right)\right)}{\left(\mathrm{O}{\mathrm{R}}_{XZ}r+g\left(r,s\right)\right)\left(\mathrm{O}{\mathrm{R}}_{ZY}r+g\left(r,s\right)\right)}, $$

where $g\left(r,s\right)=\left(1-r\right)\left(s\left(\mathrm{O}{\mathrm{R}}_{ZY}-1\right)+1\right)$. By the chain rule,

$${f}_s\left(r,s\right)=\frac{\begin{array}{c}\left(\mathrm{O}{\mathrm{R}}_{XZ}-1\right)\left(\mathrm{O}{\mathrm{R}}_{ZY}-1\right)r{g}_s\left(r,s\right)\\ \times\left(\mathrm{O}{\mathrm{R}}_{XZ}\mathrm{O}{\mathrm{R}}_{ZY}{r}^2-g{\left(r,s\right)}^2\right)\end{array}}{{\left(\mathrm{O}{\mathrm{R}}_{XZ}r+g\left(r,s\right)\right)}^2{\left(\mathrm{O}{\mathrm{R}}_{ZY}r+g\left(r,s\right)\right)}^2}.$$

Because ${g}_s\left(r,s\right)=\left(1-r\right)\left(\mathrm{O}{\mathrm{R}}_{ZY}-1\right)>0$, the partial derivative ${f}_s\left(r,s\right)$ is zero on the curve $g\left(r,s\right)=\sqrt{\mathrm{O}{\mathrm{R}}_{XZ}\mathrm{O}{\mathrm{R}}_{ZY}}r$, where $f\left(r,s\right)$ is actually a constant:

\begin{align*} f\left(r,s\right)&\le \frac{\left(\mathrm{O}{\mathrm{R}}_{XZ}\mathrm{O}{\mathrm{R}}_{ZY}\!+\!\sqrt{\mathrm{O}{\mathrm{R}}_{XZ}\mathrm{O}{\mathrm{R}}_{ZY}}\right)\left(1\!+\!\sqrt{\mathrm{O}{\mathrm{R}}_{XZ}\mathrm{O}{\mathrm{R}}_{ZY}}\right)}{\left(\mathrm{O}{\mathrm{R}}_{XZ}+\sqrt{\mathrm{O}{\mathrm{R}}_{XZ}\mathrm{O}{\mathrm{R}}_{ZY}}\right)\left(\mathrm{O}{\mathrm{R}}_{ZY}\!+\!\sqrt{\mathrm{O}{\mathrm{R}}_{XZ}\mathrm{O}{\mathrm{R}}_{ZY}}\right)}\\& ={\left(\frac{\sqrt{\mathrm{O}{\mathrm{R}}_{XZ}\mathrm{O}{\mathrm{R}}_{ZY}}+1}{\sqrt{\mathrm{O}{\mathrm{R}}_{XZ}}+\sqrt{\mathrm{O}{\mathrm{R}}_{ZY}}}\right)}^2. \end{align*}

Theorem 9. Under Assumption 1*,*

$$ \mathrm{O}{\mathrm{R}}_{XY}\le \frac{1+\left(\mathrm{R}{\mathrm{R}}_{XZ}-1\right)\mathrm{O}{\mathrm{R}}_{ZY}}{\mathrm{R}{\mathrm{R}}_{XZ}}. $$

Proof. Denote $\mathbb{P}\left(Z|\overline{X}\right)=r$ and $\mathbb{P}\left(Y|\overline{Z}\right)=s$, which implies

$$\left\{\begin{array}{ll}\mathbb{P}\left(Z|X\right)& =r\mathrm{R}{\mathrm{R}}_{XZ},\quad0<r<\frac{1}{\mathrm{R}{\mathrm{R}}_{XZ}},\\ {}\mathbb{P}\left(Y|Z\right)& =\frac{s\mathrm{O}{\mathrm{R}}_{ZY}}{s\left(\mathrm{O}{\mathrm{R}}_{ZY}-1\right)+1},\quad0<s<1.\end{array}\right.$$

According to equation 4 we can write

$$ \mathrm{O}{\mathrm{R}}_{XY}=f\left(r,s\right)=\frac{g\left(\mathrm{R}{\mathrm{R}}_{XZ}r,s\right)}{g\left(r,s\right)}, $$

where

$$ g\left(r,s\right)=\frac{\mathrm{O}{\mathrm{R}}_{ZY}r+\left(1-r\right)\left(s\left(\mathrm{O}{\mathrm{R}}_{ZY}-1\right)+1\right)}{r+\left(1-r\right)\left(s\left(\mathrm{O}{\mathrm{R}}_{ZY}-1\right)+1\right)}. $$

Differentiate

\begin{align*} {g}_r\left(r,s\right)&\!=\frac{\left(\mathrm{O}{\mathrm{R}}_{ZY}-1\right)\left(s\left(\mathrm{O}{\mathrm{R}}_{ZY}-1\right)+1\right)}{{\left(r+\left(1-r\right)\left(s\left(\mathrm{O}{\mathrm{R}}_{ZY}-1\right)+1\right)\right)}^2}>0,\\ {g}_{rr}\left(r,s\right)&\!=\frac{2\left(\mathrm{O}{\mathrm{R}}_{ZY}\!-\!1\right)\!\left(s\left(\mathrm{O}{\mathrm{R}}_{ZY}\!-\!1\right)\!+\!1\right)s\!\left(\mathrm{O}{\mathrm{R}}_{ZY}\!-\!1\right)}{{\left(r+\left(1-r\right)\left(s\left(\mathrm{O}{\mathrm{R}}_{ZY}-1\right)+1\right)\right)}^3}>0. \end{align*}

It follows that ${f}_r\left(r,s\right)>0$ and so we set $r=1/\mathrm{R}{\mathrm{R}}_{XZ}$. To maximize over *s* we differentiate

\begin{align*} {f}_s&\left(\frac{1}{\mathrm{R}{\mathrm{R}}_{XZ}},s\right)\\&=\frac{\mathrm{O}{\mathrm{R}}_{ZY}\left(1-\mathrm{R}{\mathrm{R}}_{XZ}\right){\left(1-\mathrm{O}{\mathrm{R}}_{ZY}\right)}^2}{{\left(\left(\mathrm{R}{\mathrm{R}}_{XZ}\!-\!1\right)\left(\mathrm{O}{\mathrm{R}}_{ZY}\!-\!1\right)s\!+\mathrm{\!R}{\mathrm{R}}_{XZ}\!+\!\mathrm{O}{\mathrm{R}}_{ZY}\!-\!1\right)}^2}>0 \end{align*}

and conclude that

$$ \mathrm{O}{\mathrm{R}}_{XY}\le f\left(\frac{1}{\mathrm{R}{\mathrm{R}}_{XZ}},1\right)=\frac{1+\left(\mathrm{R}{\mathrm{R}}_{XZ}-1\right)\mathrm{O}{\mathrm{R}}_{ZY}}{\mathrm{R}{\mathrm{R}}_{XZ}}. $$

## APPLICATION

As an illustrative example we use a systematic review by Owen et al. (11) on the effect of breastfeeding on type 2 diabetes; see also Ip et al. (12). Owen et al. report an aggregate odds ratio of $0.61$ (95% confidence integral: $0.44,0.85$, *P* value = 0.003) between ever breastfed vs. exclusively formula fed and later type 2 diabetes of the child. The meta-analysis comprises 7 individual studies on Europeans and Americans, one of which has a case-control design, with others based on a cohort. Because type 2 diabetes is common and underdiagnosed, converting odds ratios into risk ratios introduces complications for both cohort and case-control designs, so basing sensitivity analysis on Cornfield’s inequalities 1 or Theorems 4–7 is not advised. Instead we can use Theorems 3, 8, and 9.

To satisfy Assumption 1, we switch the order of the 2 feeding groups so that the aggregate odds ratio becomes $1/0.61=1.63$. We then ask: Can some third unmeasured binary variable *Z* fully explain the observed association between breastfeeding and the child’s type 2 diabetes status? According to Theorem 3, the association between *Z* and breastfeeding and the association between *Z* and child’s type 2 diabetes would both need to have an odds ratio of at least 1.61. Moreover, if one of the associations is weak, the other has to compensate by being strong.

Say *Z* is type 2 diabetes of the mother (diagnosed before or after the childbirth). According to Meigs et al. (13), the association between the mother’s and the child’s type 2 diabetes has an odds ratio of 3.4. By solving for $\mathrm{R}{\mathrm{R}}_{XZ}$using Theorem 9 with $\mathrm{O}{\mathrm{R}}_{XY}=1.61$, $\mathrm{O}{\mathrm{R}}_{ZY}=3.4$ we see that the risk of type 2 diabetes among mothers who do not lactate would have to be at least $1.35\dots$ times the risk among mothers who do not. Diabetics and future diabetics can have on average more trouble lactating, and lactating can have a protective effect against diabetes. A recent meta-analysis by Pinho-Gomes et al. (14) estimates that never vs. ever lactation is associated with maternal type 2 diabetes with risk ratio at $1/0.73=1.36\dots$. (We should point out that there is a slight discrepancy because a woman can have several children and only breastfeed some of them.) A noncausal association between breastfeeding and a child’s type 2 diabetes, entirely confounded by maternal type 2 diabetes, looks plausible at the moment.

However, from the proof of Theorem 9, we can see that the bound is sharpest when $\mathbb{P}\left(Z|\overline{X}\right)$, the prevalence of type 2 diabetes among women who ever lactated, is large (close to 0.73). We can reasonably argue that it should not be more than the prevalence among men, which should be safe to bound under 30%. Thus, the association reported by Pinho-Gomes et al. (14) has an odds ratio no more than 1.63, where we have used the conversion formula

$$ \mathrm{O}{\mathrm{R}}_{XZ}=\frac{\left(1-\mathbb{P}\left(Z|\overline{X}\right)\right)\mathrm{R}{\mathrm{R}}_{XZ}}{1-\mathbb{P}\left(Z|\overline{X}\right)\mathrm{R}{\mathrm{R}}_{XZ}} $$

and the crude estimate $\mathbb{P}\left(Z|\overline{X}\right)\le 0.3$. If we plug $\mathrm{O}{\mathrm{R}}_{XY}=1.61$, $\mathrm{O}{\mathrm{R}}_{ZY}=3.4$ into Theorem 8 and solve for $\mathrm{O}{\mathrm{R}}_{XZ}$, we see that the odds ratio would have to be at least 5.7 if confounding by maternal type 2 diabetes were to fully explain the observed breastfeeding-diabetes association.

Indeed, 3 of the 7 studies accounted in the meta-analysis by Owen et al. (11) used a number of relevant covariables, including parental type 2 diabetes, and when restricting to those 3 studies the aggregate odds ratio was similar ($1/0.55=1.81$) with and without covariates.
